# Effectiveness of the Cunningham technique for shoulder dislocation reduction and its role in providing analgesia and muscle relaxation as an adjunctive method

**DOI:** 10.1016/j.clinsp.2024.100447

**Published:** 2024-07-18

**Authors:** Fatih Ugur, Mehmet Albayrak

**Affiliations:** a Kastamonu University School of Medicine, Department of Orthopaedics and Traumatology, Kastamonu, Turkey; b Istanbul Rumeli University, Istanbul, Turkey

**Keywords:** Analgesia, Anterior shoulder dislocations, Cunningham technique, Muscle relaxation

## Abstract

**Background:**

Shoulder dislocation, particularly anterior dislocation, is a common orthopedic injury often presenting in emergency care settings, characterized by significant pain and muscle spasms. Prompt reduction is essential to alleviate symptoms and restore function. The Cunningham technique employs gentle pulling and massage motions targeted at the muscles and has emerged as a promising method for reducing anterior shoulder dislocations. However, its reported success rates vary widely across studies, and questions remain regarding its efficacy, particularly in cases of failure. This study aims to evaluate the effectiveness of the Cunningham technique for reducing anterior shoulder dislocations and its potential role in providing analgesia and muscle relaxation as an adjunctive method.

**Methods:**

A retrospective study was conducted on patients presenting with acute anterior shoulder dislocation at a single center. Reduction using the Cunningham technique was performed initially, followed by the external rotation technique if unsuccessful. Procedural sedation and analgesia were administered if the reduction was still not achieved, and shoulder dislocation reduction was performed again through the external rotation method. The patients’ VAS scores were recorded and evaluated the Cunningham technique's effectiveness in reduction and whether it increases the effectiveness of other techniques applied for reduction by lowering the VAS score, even in cases where it is not effective.

**Results:**

A total of 61 patients were included in the study. The reduction was performed using the Cunningham technique in 34.4% (21/61) patients, the external rotation technique in 47.5% (29/61) patients, and the external rotation technique with PSA in 18% (11/61) patients. Significant differences were observed in the duration of hospital stay among the three techniques, with ER with PSA resulting in the longest stay. VAS scores showed significant improvements from initial presentation to post-reduction in all three groups. A significant decrease in pre-reduction VAS scores was observed during the transition from the Cunningham technique to other techniques.

**Conclusion:**

The Cunningham technique showed effectiveness in reducing anterior shoulder dislocations, providing analgesia, and muscle relaxation. It demonstrated favorable outcomes as an initial reduction technique, with the external rotation technique used as a subsequent option. Further studies comparing the success rates and complications of the Cunningham technique with other reduction methods are warranted to establish its role in clinical practice.

## Highlights

• Cunningham technique; sedation-free effective reduction.
• Shorter hospital stays compared to those requiring procedural sedation for patients.
• Can be applied outdoors, reducing delays in treatment and improving outcomes.

## Introduction

### Background and importance

Shoulder dislocation is the most common joint dislocation observed in patients seeking emergency care. It typically presents as an anterior dislocation.^1^^,^^2^ There are regional variations in its incidence.^2^ Anterior dislocations of the shoulder occur due to a combination of abduction and external rotation forces applied to the arm.^2^, ^3^, ^4^ Significant demographic risk factors include male sex and age less than 30 years.^5^^,^^6^ However, shoulder dislocation is more prevalent in females aged 70–79 living in rural areas and those aged 80 or above living in urban areas.^7^

Over 50 techniques have been described for the treatment of shoulder dislocations. These are categorized as traction-countertraction techniques, leverage techniques, and biomechanical techniques.^8^ Each technique offers distinct advantages and limitations.^9^^,^^10^ The goal of employing these techniques is to promote the relaxation of the shoulder muscles and to correct the dislocated position of the humeral head.^11^

It has been demonstrated that muscle spasms are particularly important for both pain management and dislocation reduction.^8^^,^^11^^,^^12^ It is necessary to overcome muscle spasms by applying sufficient durations of traction-based techniques^13^ or through maneuvers without applying traction-based techniques (leverage-based techniques).^2^

Additionally, biomechanical techniques involve direct muscle relaxation applied to the shoulder muscles without applying any force.

### Goals of this investigation

The techniques belonging to this group include the Cunningham technique (C), the Scapular Manipulation Technique (SMT), and the Milch technique.^8^ C stands out among these and other shoulder reduction techniques as it directly separates the shoulder muscles (trapezius, deltoid, and biceps muscles) through massage without positioning the patient or the affected arm so as not to increase the pain. It was described by Neil Cunningham in 2003, and it is quick, painless, and applicable without the need for analgesics or sedation.^14^^,^^15^ While a few studies (small case series) have reported a 100% success rate for this technique,^16^^,^^17^ Puha et al. reported only a 76.9% success rate,^18^ and Campbell et al., only 35%.^11^In most relevant studies, the sedation and analgesic–free state described in the technique was not applied.^8^^,^^11^^,^^16^^,^^17^^,^^19^

The aim of the present study was to evaluate the success of C and its impact on other techniques in cases of failure.

## Methods

### Study design and setting

The patients who presented to the Emergency Department of Kastamonu Education and Research Hospital between January 1, 2017 and January 1, 2021 with acute anterior shoulder dislocation and who underwent reduction using C were included in the present retrospective study.

### Ethical approval

The study was approved by the Kastamonu University Ethics Committee (reference number: 2023-KAEK-45). Hospital records and electronic data were reviewed and analyzed to evaluate the effectiveness of C in reducing acute anterior shoulder dislocations.

### Selection of participants and outcomes measures

A diagnosis of shoulder dislocation was made based on direct radiography and clinical evaluation for all the patients.

Age, sex, side of the dislocated shoulder, length of hospital stay, Visual Analog Scale (VAS) score (0 = No pain; 10 = Extremely severe pain), prereduction and postreduction complications, and neurovascular examination findings were recorded for all the patients.

Patients were excluded if they had multi-trauma; if they had hemodynamic instability (as they could not sit in the position required); if the dislocation was accompanied by a fracture of the humerus, scapula, or clavicle; or if the dislocation had been present for more than 24h due to the known difficulties in reducing such dislocations and the potential need for sedation during the initial reduction attempt.^20^ Patients with a history of sedative/anxiolytic/analgesic/muscle relaxant use prior to hospital presentation were also excluded.

All patients who met the study criteria and were admitted for treatment were initially subjected to C for 3 min. This duration was set as 75% of the patients in the initially described C technique experienced shoulder dislocation reduction after being subjected to C for 3 min or less.^14^ If success was not achieved using this method, the External Rotation technique (ER) was employed. If success was still not achieved with this method, Procedural Sedation and Analgesia (PSA) was applied, and shoulder dislocation reduction was performed again through the external rotation method (ER with PSA).

C and ER were performed by senior orthopedic surgeons who were instructed about the proper use of these two methods without the administration of any sedative, muscle relaxant, anxiolytic, or analgesic medications. Initially, C was applied to all the patients. In cases where reduction was not successful with this technique, ER was used. If reduction could also not be achieved with ER, the reduction was performed and concluded with ER with PSA by the same physician. Neither in C nor in ER were intra-articular injection and peripheral nerve block used.

ER was chosen for use in the present study to modify the effects of muscle relaxation provided by C, ensure a sufficient muscle length for the pectoralis major, and influence shoulder stability.^21^ In our practice, massage of the pectoralis major muscle is initiated, along with the application of ER.

As indicated in Labriola et al.,^21^ the pectoralis major muscle has both enhancing and destabilizing effects on shoulder stability. Therefore, as soon as the transition to ER is made to increase the elasticity and length of the pectoralis major, massage of the pectoralis major muscle is also started.

In cases where anterior shoulder dislocation reduction via the Cunningham technique failed after two attempts, sedation, primarily with midazolam (2 mg or 0.05 mg/kg) or occasionally propofol (1–2 mg/kg), was administered under the supervision of the physician performing the reduction maneuver in the emergency department, ensuring patient monitoring during the procedure.^10^^,^^18^ The technique performed with PSA was ER. After reduction, all the patients were immobilized with a Velpau bandage. They were then followed up at the end of the first and third weeks postreduction.

The length of hospital stay was determined by examining the timestamps of the radiographs taken before and after the reduction procedure while accounting for factors such as transportation time between radiographs to mitigate any potential bias related to hospital efficiency. In the cases where PSA was administered, the patients were monitored until stable, and after an appropriate waiting period for the medication to take effect, the reduction occurred, and radiographs were taken thereafter.^10^

The patients' Visual Analog Scale (VAS) Scores were assessed upon their initial presentation to the emergency department, before the application of each reduction technique, during the mid-reduction phase, and upon completion of the reduction procedure. Statistical analysis was conducted to examine for any significant differences among these scores. Before the application of ER to the patients in which reduction was not achieved with C, VAS score measurements were conducted to determine the changes in pain after the completion of C. However, for the patients who did not achieve a reduction despite the use of C and ER, PSA was administered. Thus, an assessment in the phase During Reduction (DR) could not be performed.

### Reduction methods

C is performed as follows:

1) The patient is seated upright in a chair or on a patient bed, and the importance of their cooperation for a successful reduction is explained.
2) The clinician positions themself beside the patient, either kneeling or sitting and places their wrist on the patient's forearm on the affected side, with their hand resting on the elbow between the body and the arm.
3) During this phase, the clinician refrains from applying downward traction on the affected arm to prevent muscle spasms.
4) The trapezius, deltoid, and biceps muscles are massaged repeatedly, with the process repeated several times.
5) Once the arm is completely relaxed, the humeral head is rapidly and painlessly relocated to achieve reduction. It is important to note that the traditional audible “pop” sound may not always be present, requiring frequent reassessment to confirm the successful relocation of the shoulder.^9^^,^^14^

ER is performed as follows:

1) In the supine position, the patient's arm is adducted, and the clinician stands at the side of the bed.
2) The clinician holds the patient's wrist with one hand and stabilizes the elbow with the other hand.
3) The elbow is flexed at 90 degrees, and the shoulder is positioned at 2 degrees of forward flexion.
4) The clinician gradually and smoothly externally rotates the patient's arm, using the wrist as a guide, and slowly externally rotates the shoulder until reaching approximately 180 degrees Typically, the shoulder is reduced within the range of 70–110 degrees of ER. The arm is internally rotated to bring the forearm into the abduction position.^9^^,^^22^^,^^23^.

### Statistical data analysis

The distributions of the data were assessed using the Shapiro-Wilk test. In cases where the assumption of normal distribution was violated for more than two dependent groups, the Friedman test was used for comparison. Comparisons among the three independent groups were conducted using the Kruskal-Wallis test. Post hoc comparisons of significant variables were performed using the Dunn test. Categorical data were compared using the Fisher-Freeman-Halton test. Descriptive statistics for the data were reported as mean ± standard deviation or median (min–max). Descriptive statistics for categorical data were presented as frequency (percentage). All statistical analyses were conducted using the IBM SPSS Statistics 26.0 software at a significance level of α = 0.05, and the results were reported accordingly.

## Results

A total of 69 patients were initially enrolled in the study. After exclusions, 61 patients with an average age of 44.66±21.54 years were included, consisting of 17 females (27.9%) and 44 males (72.1%). Reduction procedures were performed on 35 right shoulders (57.4%) and 26 left shoulders (42.6%). The distribution of reduction techniques utilized was as follows: Cunningham technique (C) in 21 patients (34.4%), External Rotation (ER) in 29 patients (47.5%), and ER with Procedural Sedation and Analgesia (PSA) in 11 patients (18%). The mean hospital stay for all patients was 56.97±55.76 minutes (11–210).

The results of the statistical analysis comparing the patients’ demographic characteristics based on technique types are presented in Table 1.

**Table 1:** Evaluation of the patients’ demographic characteristics based on the reduction technique used.

	C^1^ (*n* = 21)	ER^2^ (*n* = 29)	ER with PSA^3^ (*n* = 11)	*p*-value^a^^,^^b^	Post hoc
**Gender**					
♀	4 (23.5%)	11 (64.7%)	2 (11.8%)	0.268^a^	‒
♂	17 (38.6%)	18 (40.9%)	9 (20.5%)		
**Side**					
*Right*	14 (40%)	17 (48.6%)	4 (11.4%)	0.315^a^	‒
*Left*	7 (26.9%)	12 (46.2%)	7 (26.9%)		
**Time in hospital (min)**	26 (11–94)	28 (14–89)	166 (115–210)	**<0.001**^b^	**1–2:1**
					**1–3:<0.001**
					**2–3:<0.001**
**Age**	28 (17–74)	39 (16–77)	48 (25–86)	0.409^b^	‒

a Fisher-Freeman-Halton exact test.

b Kruskal-Wallis test. The data were presented as frequency (percentage) or median (min–max). C, Cunningham technique; ER, External Rotation technique; PSA, Procedural Sedation and Analgesia.

In Table 1, the patients’ ages, shoulders operated on, and duration of hospital stay were statistically compared by reduction technique. No significant differences were found based on sex, shoulder operated on, age, experience (C, ER), and use of ER with PSA (*p* > 0.05). However, there were significant differences in the duration of hospital stay (in minutes) by applied technique (*p* < 0.001). According to the post hoc comparison, there was no significant difference in the duration of hospital stay between C and ER (*p* = 1), indicating their similarity. However, there were significant differences in the duration of hospital stay between the ER-with-PSA, C, and ER groups (*p <* 0.001). The patients who were sedated also had significantly longer hospital stays than those who were manipulated with C and ER.

**Fig. 1:**
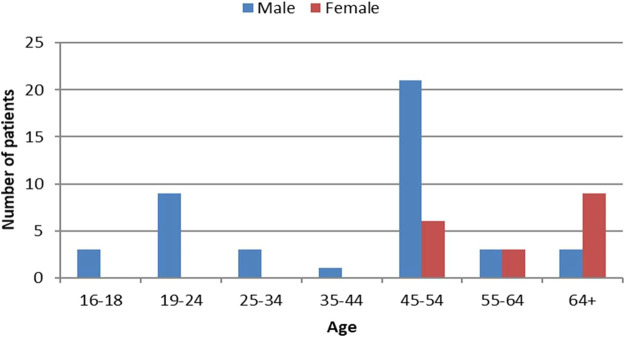
Age and gender distribution of the patients.

The age and sex distributions of the patients are shown in Fig. 1.

The analysis results of the differences between the Initial Presentation (IP), Before-Reduction (BR), During-Reduction (DR), and Postreduction (PR) VAS scores within each patient group are presented in Table 2.

**Table 2:** Initial presentation, before-reduction, during-reduction, and postreduction visual analog scale scores.

Group	Initial presentation^1^	Before reduction^2^	During reduction^3^	Postreduction^4^	*p*-value^a^	Post hoc
C	8 (6–9)	8 (6–9)	3 (2–4)	2 (1–3)	<0.001	1–2:1
						1–3:<0.001
						1–4:<0.001
						2–3:<0.001
						2–4:<0.001
						3–4:0.0335
ER	7 (6–9)	5 (4–6)	3 (2–4)	2 (1–3)	<0.001	1–2:0.019
						1–3:<0.001
						1–4:<0.001
						2–3:0.008
						2–4:<0.001
						3–4:0.088
ER with PSA	8 (7–9)	6 (5–6)	-	2 (2–3)	<0.001	1–2:0.057
						1–4:<0.001
						2–4:0.057

a Friedman test. The data were presented as median (min–max).

**Fig. 2:**
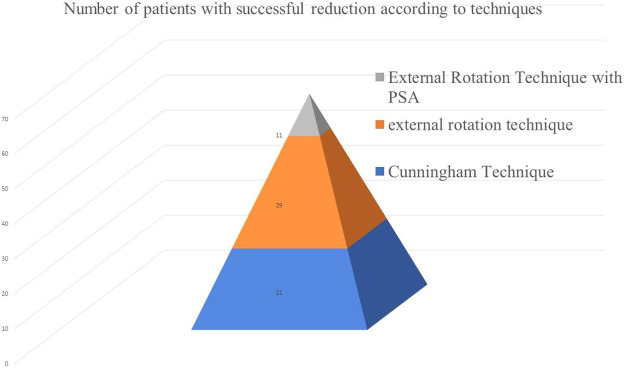
Patient distribution according to reduction technique.

Significant differences were found in the VAS scores at IP, BR, DR, and PR in the C, ER, and ER-with-PSA groups (*p <* 0.001). Post hoc comparisons for C and ER revealed significant differences in the IP–DR, IP–PR, and DR–PR comparisons (*p <* 0.05). There were no significant changes in the IP–BR values in the C and ER-with-PSA groups, but there were statistically significant changes (1–2:0.019) in ER. In the cases in which C failed and ER was applied, significant changes were observed in the BR values of ER. However, there were insufficient changes in the values of the patients who underwent PSA before reduction. The IP VAS score was significantly higher than the DR and PR VAS scores, and the DR VAS score was significantly higher than the PR VAS score. In ER with PSA, a significant difference was found in the IP–PR VAS scores, where the IP VAS score was significantly higher than the PR VAS score. The IP–DR and DR–PR VAS scores were not evaluated because the patients were under sedation. Fig. 2 illustrates the patient distribution by reduction technique.

The statistical analysis results comparing the changes in VAS scores in the groups subjected to the three aforementioned reduction techniques compared to the baseline are presented in Table 3.

**Table 3:** Comparison of the changes in visual analog scale (VAS) scores relative to baseline in the groups subjected to the three reduction techniques.

	C^1^	ER^2^	ER with PSA^3^	*p*-value^a^	Post hoc
**VAS Δ1**	-5 (-7:-3)	-4 (-6:-3)	‒	0.062	‒
**VAS Δ2**	-6 (-8:-4)	-5 (-8:-4)	-6 (-7:-4)	0.071	‒
**VAS Δ3**	0 (0:0)	-2 (-4:-1)	-2 (-4:-1)	**<0.001**	**1–2:<0.001**
					**1–3:<0.001**
					2–3:1

Δ1: Initial presentation – during reduction.

Δ2: Initial presentation – postreduction.

Δ3: Initial presentation – before reduction.

a Kruskal- Wallis test. The data were presented as median (min–max).

**Fig. 3:**
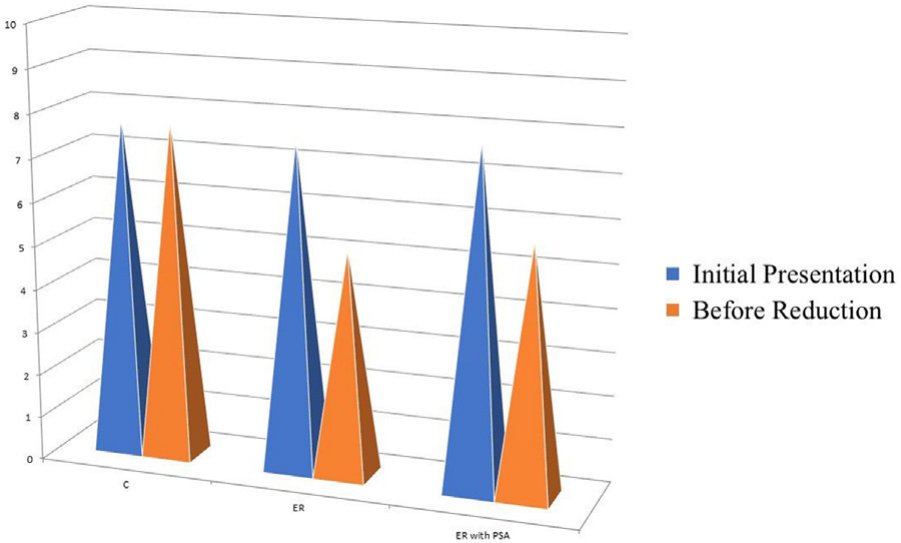
Distribution of the VAS scores by reduction method.

In Table 3, the changes in the VAS scores from the Baseline (BR) after the application of the three reduction techniques are compared. The changes showed statistically significant differences (*p <* 0.001). The post hoc test indicated that there were differences between the C and ER groups and between the C and ER-with-PSA groups. It was concluded that the change in pain was more pronounced in the ER and ER-with-PSA groups than in the C group. However, there were no statistically significant differences in the change in VAS scores from the Baseline (BR) in the ER and ER-with-PSA groups. There were also no statistically significant differences in the change in DR VAS scores from the Baseline (BR) between the C and ER groups or between the ER and ER-with-PSA groups (*p >* 0.05). No statistically significant differences in the change in VAS score after reduction from the baseline were shown between the groups (*p =* 0.071). Fig. 3 illustrates the distribution of the VAS scores by the reduction method.

**Fig. 4:**
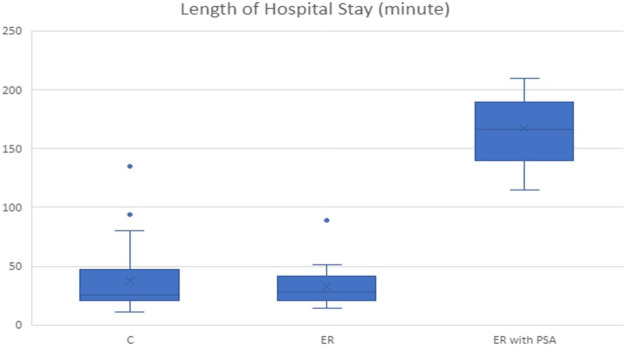
Distribution of the time spent during the different reduction maneuvers.

No complications were experienced during the BR or PR periods. The neurovascular evaluations of all the patients were recorded as normal both before and after reduction. Fig. 4 illustrates the distribution of the time spent during the different reduction maneuvers.

## Discussion

In this retrospective study, C was described as an effective treatment method for anterior shoulder dislocation, as originally defined, without the need for PSA, intra-articular injection, or peripheral nerve block. Even if it does not achieve reduction, it is still useful, having been reported to decrease pain and relax the muscles, resulting in increased patient comfort for the other shoulder dislocation reduction methods to be used.

Some authors state that the ideal approach to shoulder dislocation reduction involves ensuring sufficient muscle relaxation and analgesia under general anesthesia.^12^^,^^24^ However, this approach is not the preferred one due to the potential side effects associated with the drugs used, the need for monitoring, prolonged hospital stays increased need for healthcare personnel throughout the process, and the resulting increase in costs.^2^^,^^10^^,^^22^

Many techniques for shoulder dislocation reduction other than C have been described. These techniques involve the use of traction and/or rotation combinations.^9^ There is no clear and full explanation for why shoulder dislocation reduction occurs or fails to occur in these techniques.^25^ Rarely, patients can even achieve spontaneous reduction by themselves.^26^ While some studies have suggested the influence of the shoulder bone structure (especially glenoidal version and inclination) on reduction,^27^ the key factor for successful reduction is sufficient relaxation in the known shoulder muscles.^1^^,^^11^^,^^13^, ^14^, ^15^^,^^28^, ^29^, ^30^

Muscle spasms in shoulder dislocation are characterized by the simultaneous involvement of multiple muscles. The occurrence of muscle spasms triggers a cascade of events, including muscle ischemia, leading to a decrease in pH levels and the release of pain-inducing substances such as bradykinin, ATP, and H+. This establishes a vicious circle mechanism in which muscle pain initiates spasms, which exacerbate the pain experience, leading to the need for more aggressive measures and PSA.^31^

Furthermore, it is important to note that muscle spasms can be induced by pain originating from adjacent muscles. For instance, studies have reported spasm-like increased electromyographic activity in the trapezius muscle in response to painful stimulation of the biceps brachii muscle.^32^ Additionally, pathological alterations in neighboring joints can contribute to the occurrence of muscle spasms. It is crucial to actively investigate these potential sources of pain and address them accordingly.

In shoulder dislocation, multiple muscle spasms occur, and these spasms not only trigger spasms in other muscles but also make reduction by the traction-countertraction methods more difficult due to pathological changes in the shoulder joint. However, the C technique, which reduces muscle spasms, facilitates reduction. In cases where reduction cannot be achieved, other reduction methods can be easily applied because the muscle spasm has already been resolved.

The aim of the C technique is to reduce dislocation by directly massaging the deltoid, biceps brachii,^21^^,^^33^ and trapezius muscles,^32^ which are effective muscles for glenohumeral joint stability, thereby reducing pain and inducing muscle relaxation.^14^^,^^15^^,^^30^ Studies that include the C technique mostly consist of a small number of cases,^14^^,^^16^^,^^17^^,^^34^ have specific technical descriptions,^2^^,^^9^^,^^10^^,^^15^^,^^22^ and involve sedation and/or analgesic use.^8^^,^^11^^,^^14^^,^^16^^,^^19^^,^^30^^,^^35^ Our study is one of the largest case series in the literature that utilized the C technique as a reduction method without the use of analgesia and sedation.

The technique defined as drug-free by Neil Cunningham has been studied with various applications and success rates. In the study by Campbell et al.,^11^, methoxyflurane inhalation achieved a 35% success rate in only 20 patients. Baden et al.^8^ reported a 23% success rate in 43 patients, with 58% receiving Intravenous (IV) medication and 44% receiving oral medication. Walsh et al.^16^ and Mati et al.^17^ used intra-articular medication in only two out of three patients. Gudmundsson et al.^35^ applied the C technique to one patient in their first year they started to use the C, reducing sedation use to 73%, and achieved a 28.5% success rate in 28 patients in their second year. Batur et al.^36^ reported a 100% success rate in 6, 14, and 10 patients, respectively, when the C technique was applied by three clinicians, with fentanyl used for analgesia. Stoesz et al.^34^ did not specify the drugs used in the C technique but applied traction with massage.

An interesting aspect of the C technique is that the largest case series includes ski patrollers who have been trained in the technique. Using only the C technique, ski patrollers achieved an impressive 74.3% success rate, with 83% using nitrous oxide in the process.^19^ In this study, it was observed that the patient's arms were flexed in a manner not described in the classical C technique. Although the applied massage describes the C technique in terms of pain, when the patient's position is considered, it resembles the Sool technique. In this technique, the patient's hand is placed on the practitioner's shoulder, and gentle traction is applied with massage, targeting only the deltoid anterior fibers and pectoralis major muscles. It must be noted that changing the position of the affected arm in such an application may increase the pain. Following Cunningham's initial report, the only drug-free study the authors encountered was conducted by Puha et al.^18^ They reported a 76.9% success rate for the C technique in their study involving 50 patients, in which they employed four different techniques.

To our knowledge, the present study was the largest case series in terms of the C technique performed by the same physician on consecutive patients. A 34.4% success rate was achieved by applying the C technique to all patients without using any medications.

The importance of the C technique lies in its ability to achieve muscle relaxation and an analgesic effect through massage without requiring patients to assume any specific position, such as supine or decubitus, or without applying traction. Other techniques have been observed to have a tendency to increase pain and muscle spasms. This can be seen particularly in the evaluation of the VAS scores during reduction. Comparisons were made between the results of our study and those of Baden et al.’s and Batur et al.’s studies due to similarities in the initial VAS values.^8^^,^^37^ When Baden et al. applied the C technique, they found that the VAS score was 5.0, while in the modified Milch technique, it was 6.1, and in the SMT technique, it was 5.9.^8^ On the other hand, Batur et al.^37^ found a VAS score of 8 (7–9) for the traction-countertraction technique, 5 (4–7) for the external traction technique, and 4 (2.75–5) for the C technique. In the present study, the DR VAS score in the C technique was 3 (2–4), and the PR VAS score was 2 (1–3), which is similar to Batur et al.’s findings.^37^ Thus, it can be said that the C technique effectively breaks the vicious cycle of pain and muscle spasms by significantly lowering the DR VAS scores. During the technical application, pain reduction should be considered evidence.

In terms of unsuccessful cases, to the best of our knowledge, the lowest initial VAS score for other techniques was reported to be 5 (4–6), which is the BR VAS score. In the present study, the second technique applied was ER, with a 47.5% success rate among all the patients. The overall success rate for both techniques was 82%. Eachempati et al.^38^ achieved a reduction without requiring any premedication in 72.5% of the cases using ER in their study. The authors achieved a similar success rate with ER (72.5%), excluding the C method.

Janitzky et al.^28^ found a 78.7% success rate, which increased to 88.1% when sedation was applied. In this study, the length of hospital stay was 55±17 min in the group without sedation and analgesia, while it increased to 118 ± 23 min in the sedated group. When comparing hospital stays, Baden et al.^8^ reported 125 min for the C/modified Milch technique, Gudmundsson et al.^35^ reported 219 min, and Campbell et al.^11^ reported 149 min. It should be noted that sedation or a history of IV medication use influenced these results. In the present study, the hospital stays were 26 (11–94) min for C, 28 (14–89) min for ER, and 166 (115–210) min for the sedation group. There were significant differences from the results of other studies. Although some studies have suggested that the C technique is not effective due to its low success rate in emergency departments,^39^ the length of hospital stay for the C technique in the present study was the opposite. In the treatment of shoulder dislocation, the longer the delay, the more unsuccessful the reduction attempts;^40^ thus, C is a safe technique that can be applied not only in medical settings but also at the initial scene or during transfers, either by healthcare professionals or educated volunteers who have been trained in this matter. It has the advantage of reducing pain, providing muscle relaxation, and enhancing patient comfort. The C technique, both by itself and compared with other techniques, helps reduce the hospital stay duration. As seen in the present study, if sedation is taken into consideration, the hospital stay duration is similar to that in other studies.

With regard to the success rate of the C technique, no increase or decrease in it over the years was observed in the present study.

## Conclusion

The Cunningham technique demonstrates notable effectiveness in reducing anterior shoulder dislocations. Its advantages lie in its ease of application, even by non-healthcare professionals with adequate training, and its potential to initiate treatment upon successful reduction. Furthermore, while the technique may not always achieve reduction, it aids in alleviating pain and muscle spasms, facilitating subsequent reduction methods.

## Ethical statement

The study was approved by the Kastamonu University Ethics Committee (reference number: 2023-KAEK-45).

## Financial support

None.

## CRediT authorship contribution statement

**Fatih Ugur:** Methodology, Formal analysis, Visualization, Writing – review & editing. **Mehmet Albayrak:** Conceptualization, Data curation, Methodology, Formal analysis, Visualization, Writing – original draft, Writing – review & editing.

## Declaration of competing interest

The authors declare no conflicts of interest.
